# Combined skin and muscle vaccination differentially impact the quality of effector T cell functions: the CUTHIVAC-001 randomized trial

**DOI:** 10.1038/s41598-017-13331-1

**Published:** 2017-10-12

**Authors:** G. Haidari, A. Cope, A. Miller, S. Venables, C. Yan, H. Ridgers, K. Reijonen, D. Hannaman, A. Spentzou, P. Hayes, G. Bouliotis, A. Vogt, S. Joseph, B. Combadiere, S. McCormack, R. J. Shattock

**Affiliations:** 1 Imperial College London, Department of Medicine, Section of Virology, Group of Mucosal Infection and Immunity, London, United Kingdom; 2 FIT Biotech Ltd., Tampere, Finland; 3 Ichor Medical Systems Inc, San Diego, CA United States; 4 Human Immunology Laboratory, International AIDS Vaccine Initiative, London, United Kingdom; 5 Clinical Research Center for Hair and Skin Science, Department of Dermatology and Allergy, Charité - Universitätsmedizin Berlin, Charitéplatz 1, 10117 Berlin, Germany; 6 Medical Research Council Clinical Trials Unit at UCL, University College London, London, UK; 7 Sorbonne Universités, UPMC Univ Paris 06, INSERM, U1135, CNRS, ERL 8255, Centre d’Immunologie et des Maladies Infectieuses (CIMI-Paris), 91 Boulevard de l’Hôpital, F-75013 Paris, France

## Abstract

Targeting of different tissues via transcutaneous (TC), intradermal (ID) and intramuscular (IM) injection has the potential to tailor the immune response to DNA vaccination. In this Phase I randomised controlled clinical trial in HIV-1 negative volunteers we investigate whether the site and mode of DNA vaccination influences the quality of the cellular immune responses. We adopted a strategy of concurrent immunization combining IM injection with either ID or TC administration. As a third arm we assessed the response to IM injection administered with electroporation (EP). The DNA plasmid encoded a MultiHIV B clade fusion protein designed to induce cellular immunity. The vaccine and regimens were well tolerated. We observed differential shaping of vaccine induced virus-specific CD4 + and CD8 + cell-mediated immune responses. DNA given by IM + EP promoted strong IFN-γ responses and potent viral inhibition. ID + IM without EP resulted in a similar pattern of response but of lower magnitude. By contrast TC + IM (without EP) shifted responses towards a more Th-17 dominated phenotype, associated with mucosal and epidermal protection. Whilst preliminary, these results offer new perspectives for differential shaping of desired cellular immunity required to fight the wide range of complex and diverse infectious diseases and cancers.

## Introduction

Tailoring of the immune responses to DNA vaccination via targeting of different tissues (epidermis, dermis and muscle) has received little attention. Differences in the density and phenotype of antigen presenting cells (APCs) appear critical in shaping the ensuing adaptive immunity, as the original antigen encounter with APCs determines subsequent imprinting of the quality of the immune response^1^. There are two main target tissues used for parenteral vaccination: the skin (epidermis and dermis) and muscle. In the absence of tissue damage or inflammation, skeletal muscle contains a relatively small population of resident immune cells^2,3^. By contrast the skin is rich in professional APCs, including epidermal Langerhans cells (LCs) and dermal dendritic cells (DCs), which are known to migrate to draining lymph nodes and trigger immune responses^4^. Infiltrating polymorphonuclear (PMN) neutrophils may also play a unique role in acting as “antigen carriers” delivering immunogens and engulfed apoptotic cells to APCs in the bone marrow^5^, influencing the quality of CD8 + T cell responses.

Intramuscular (IM) vaccination is entirely dependent upon the influx of monocytes that differentiate into APC and PMNs, whilst the uptake and transport of antigen in the skin is likely mediated by both resident and recruited APC/PMNs. Their recruitment and activation is typically triggered by an inflammatory response, which is the basis of most adjuvant strategies^6^. Plasmid DNA (pDNA) is able to induce innate activation by triggering cytoplasmic sensing through the cGAS/STING/IRF7 pathway leading to type I interferon secretion^7,8^. The extent to which different APC subsets respond to pDNA has not been fully characterised.

Muscle remains the site of choice for DNA vaccination due to its relative mass, ability to accommodate relatively large volumes of DNA and the ease of myocyte transduction. Although cellular immune responses can be induced by IM injection alone, DNA electroporation (EP) has increasingly been shown to overcome the limitations of this route in humans. EP creates an electric field at the vaccine site breaking down muscle cells and creating temporary cell membrane instability, thereby facilitating increased uptake of DNA. Importantly, the inflammation associated with EP is also thought to enhance APC recruitment^9,10^. In recent phase I HIV-1 prophylactic vaccine trials, IM DNA vaccination with EP has been shown to improve both cellular and antibody mediated responses^11,12^.

Cutaneous vaccination by skin scarification represents the oldest and most successful route of vaccination used for traditional smallpox vaccines, dependent on the generation of protective T-cell memory immune responses^13^. Surprisingly transcutaneous (TC) vaccination, despite the potential to target different APCs, has been underexplored for vaccine delivery in clinical trials. Recent work, by us and others, highlights the potential benefits of hair follicle targeting following cyanoacrylate skin surface stripping as a promising delivery technique for transcutaneous immunization^14,15^. A previous comparative clinical study using an inactivated influenza vaccine indicated preferential amplification of CD8 + effector-T cells after TC application in comparison to IM injection, comparable CD4 + responses were induced by both routes of administration, while humoral responses were induced by IM administration only^16,17^. Previous studies have shown effective DNA immunization via hair follicles following hair depilation in mice^18^. These results provide a strong rationale for adapting DNA vaccination strategies for complex infections such as HIV, influenza, hepatitis C virus and cancer, where cellular responses are considered central to controlling disease. To the best of our knowledge there is no documented evaluation of transcutaneous DNA vaccination in humans. Intradermal (ID) delivery by contrast has been extensively studied in many clinical trials of protein-based vaccines. The higher density of intradermal APC and their efficient loading can result in dose sparing with some vaccines using up to a tenth of the standard dose for intramuscular immunization^19^. Few clinical studies have directly compared the performance of the two routes for delivery of DNA but preliminary data suggests ID administration to be dose sparing and superior with or without EP^20,21^. The findings are supported by animal data suggesting that greater polyfunctional CD4 + T cell responses are seen after ID administration of a DNA vaccine even at 20% of the dose when the same vaccine is administered IM.^22^.

In this Phase I randomised controlled clinical trial we investigate whether the site and mode of DNA vaccination can influence the quality of the cellular immune responses, using a vaccine specifically designed to induce cellular and not antibody responses. In order to overcome the constraints on the volume that can be delivered via TC and ID routes (maximum of 200 μl), we employed a strategy of concurrent immunization combining IM injection (4 mg dose, IM_4_) with either ID or TC administration (0.4 mg dose, ID_0.4_ and TC_0.4_). We hypothesized that such combined vaccination might tailor the quality of the immune responses by maximizing transgene expression through IM injection, while targeting diverse APC populations mediated by TC or ID co-administration^23^. Our own studies in mice have suggested that co-administration (ID + IM) of a DNA vaccine enhanced cellular responses^24^. As a third arm we assessed the response to IM_4_ injection (full dose) administered with EP. In an attempt to maximize gene expression we used a DNA plasmid employing the gene transfer unit (GTU®) technology which utilizes the bovine papilloma virus type 1 (BPV1) transcriptional activator, the segregation/partitioning factor E2 protein, and its multimeric binding sites^25^. This has been shown to result in enhanced transcriptional activity along with the potential for increasing the number of cells expressing the transgene^26^. The same plasmid has been shown to have a modest impact on viral load when administered to HIV positive subjects in South Africa^27^. We report on the safety of this DNA vaccine in HIV-1 negative volunteers and demonstrate differential shaping of CD4 + and CD8 + cell-mediated immune responses.

## Results

### GTU^®^MultiHIV B clade DNA vaccine is well tolerated in healthy HIV negative adult volunteers when delivered by TC, ID and IM administration with and without EP

Study volunteers were self-selected members of the public responding to recruitment material that invited an expression of interest to take part in the trial. 58 participants were screened with 30 deemed eligible and enrolled according to the protocol defined inclusion and exclusion criteria (Supplemental Table 1). Three participants (one from each group) did not complete all vaccine visits: in group 1 the participant moved away before the trial was completed and only received two vaccinations; in group 2 the participant chose not to receive the third vaccine due to mild to moderate persisting hypopigmentation following TC administration of the DNA vaccine; in group 3 the participant chose not to continue with vaccinations due to moderate pain as a result of the EP after the first vaccination (Supplemental Fig. 1). This resulted in a total number of 27 volunteers completing the full trial protocol with a total of 86/90 doses of vaccine administered according to the protocol-defined groups. The majority of participants were White (77%), and male (56%). The mean age was 30.7 years (Supplemental Table 2). The immunogenicity analysis is presented per protocol (PP) and excludes the participants that did not complete the immunisations.

Overall the GTU^®^MultiHIV B clade DNA vaccine was safe and similarly tolerated across all 3 vaccination groups. Two individuals (TC, EP) chose to discontinue following adverse events, although this was not due to clinical concerns. The majority of unsolicited adverse events were unrelated to vaccination and there were no serious adverse events during the trial. From participant diary card entries capturing solicited events, there were no major differences in general symptoms across the groups (Supplemental Fig. 2). There were a greater number of local (arm) symptoms reported in the TC_0.4_ + IM_4_ group compared to the ID_0.4_ + IM_4_ group (Supplemental Table 3). Of note 5/11 participants in the TC_0.4_ + IM_4_ group reported either hypo or hyperpigmentation over the transcutaneous vaccine site, most likely a post-inflammatory phenomenon to DNA vaccination. This has not been previously observed following TC vaccination when using seasonal influenza vaccine^16^. Although all these skin changes resolved, 1 participant in this group declined the final vaccine at week 12 due to persistent hypopigmentation which started at week 4.

The EP tolerability data collected from participants receiving all three immunizations shows the maximum grade of pain reported immediately and 30 minutes after EP with the majority of responses rated as moderate and mild respectively (Supplemental Fig. 3). Although one participant declined further immunisations with EP due to pain, no-one thought that the procedure was unacceptable if it protected people from a serious disease for which no vaccine was currently available (Supplemental Fig. 4).

### Lack of humoral response to the GTU^®^MultiHIV B clade DNA vaccine

The GTU^®^MultiHIV B clade DNA vaccine was designed to induce cellular responses, encoding a fusion protein with no secretion signal. We assessed systemic and mucosal antibody responses to determine whether route of administration might influence the potential to induce humoral responses. However there was no detectable systemic or mucosal antibody response to any of the vaccine immunogens in all of the treatment groups, in line with previous studies using pDNA encoded T cell immunogens^11^.

### Electroporation preferentially expands interferon-γ T cell responses in healthy volunteers

**Figure 1:**
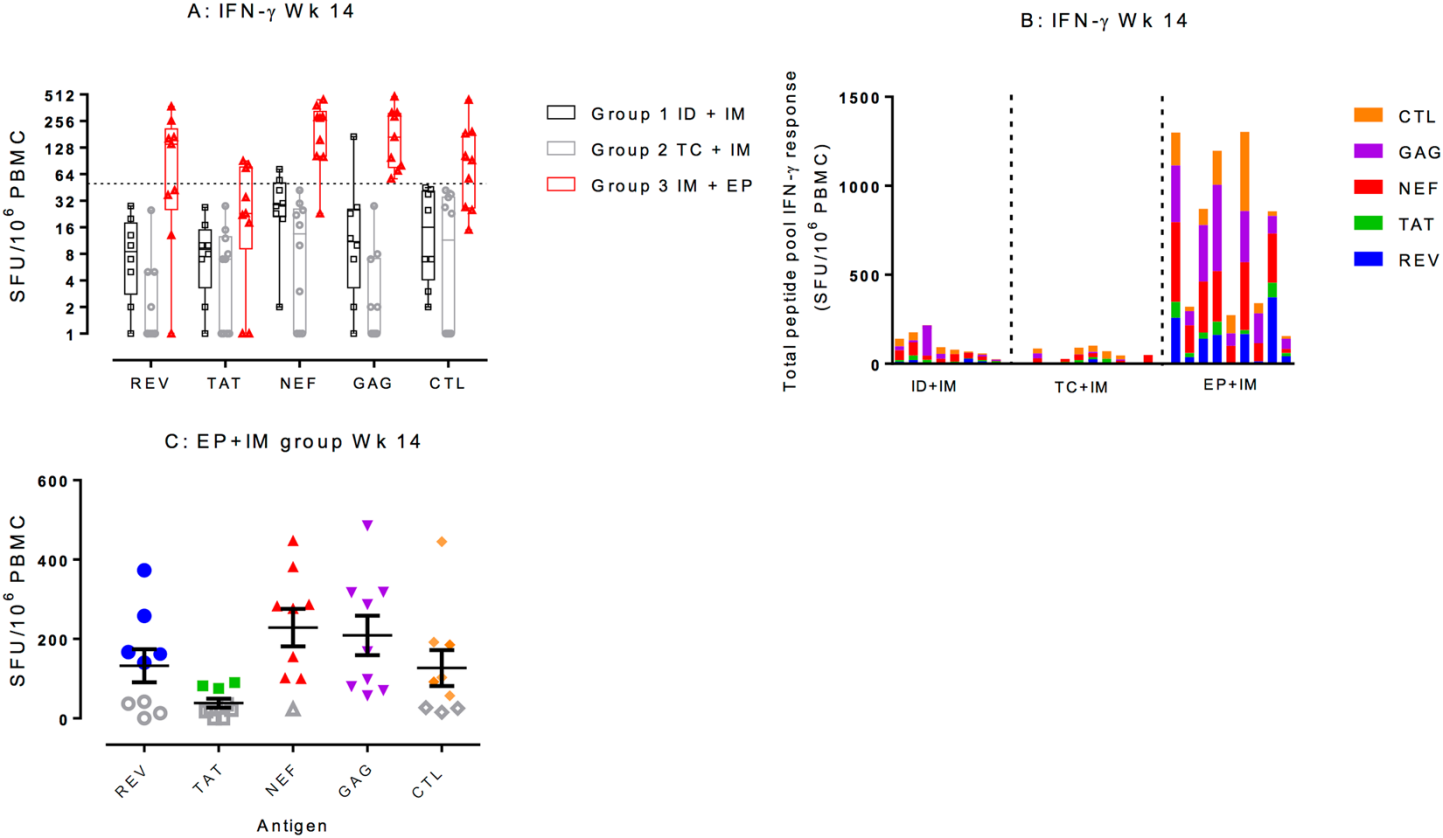
Summary of immunogenicity. (**A**) T cell IFN-ɣ ELISpot responses at the primary end point (week 14). All responses expressed as spot forming units per million PBMCs with background subtracted (SFU/M +/− SEM). The dotted line represents a ‘positive’ response defined as >55 SFU/M and at least 4 x mean background response. Box plots based upon data from responders only are superimposed on the distributions, mid-line denotes mean, ends of the box denote 25th and 75th percentiles and where whiskers that extend from the top and bottom are the extreme data points. (**B**) Total combined peptide pool response at the primary end point by group where each stacked bar represents an individual participant with each colour representing a different peptide pool. (**C**) IFN-ɣ response in EP + IM group at week 14 by peptide pool.

Positive T cell ELISpot IFN-ɣ responses at the primary end point (week 12) were seen in 1/9 participants (11%) who received ID_0.4_ + IM_4_ vaccination, and 9/10 (90%) that received EP + IM_4_ vaccination (Fig. 1A,B). There were no positive responses by IFN-ɣ in the TC_0.4_ + IM_4_ group at the primary end point. The magnitude of the IFN-ɣ response was greatest in the EP + IM_4_ group across all peptide pools, with a statistically significant difference between the groups (Supplemental Table 4). Within the EP + IM group, the greatest magnitude of IFN-ɣ response was to Nef and Gag peptide pools with geometric means of 157.0 SFU/M (95% CI, 80.7 to 305.3) and 125.4 SFU/M (95% CI, 57.5 to 273.4) respectively (Fig. 1C). The EP + IM group showed enhanced responses at weeks 14 and 20 (Supplementary Figure 5, with individual kinetics shown in Supplementary Figure 6).

### Transcutaneous vaccination modulates the quality and function of induced T cell response

**Figure 2:**
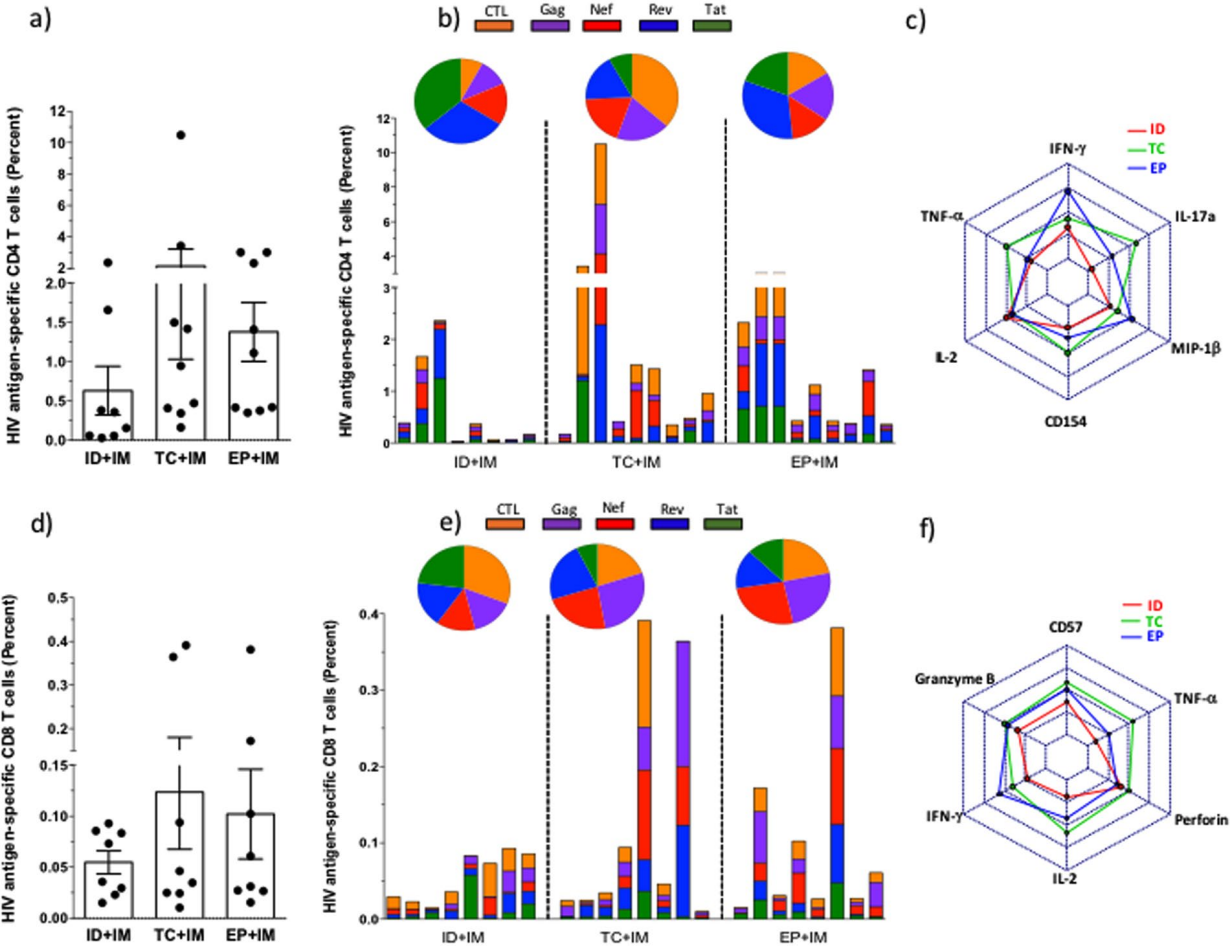
Multi-parametric ICS T cell analysis. Baseline V2 percentages have been subtracted from the V10 (primary end point) for both CD4 (**a**–**c**) and CD8 responses (**d**–**f**). The scatter plot in (**a**), (**d**) shows total antigen specific CD4 or CD8 responses (respectively) according to vaccination group measured by ICS, the boxes showing the mean +/− SEM. When analysed statistically no significant differences were seen between vaccination groups for CD4 or CD8 responses (Mann-Whitney U test. Parts (**b** & **e**). Bar charts shows each of the individual responses according to vaccination group to each of the vaccine encoded antigens as CD4 (**b**) or CD8 (**e**), the pie charts above each vaccination group demonstrate the proportion of antigen specific T cells. Radar plots (**c**) CD4 and (**f**) CD8 demonstrate the mean T cell response for each vaccination group according to cytokine expression levels as a marker of function.

To study the quality of cellular immune response we assessed the phenotype of vaccine-induced T cells by intracellular cytokine staining (ICS) stimulated with the vaccine encoded peptide pools. Analysis of the total percentage of CD4 + responses by vaccination revealed responses in all three groups with a non-significant trend for greater magnitude of response in the TC_0.4_ + IM_4_ and EP + IM_4_ groups compared to the ID_0.4_ + IM_4_ group (Fig. 2a). The breadth of CD4 + response was also greater in both TC_0.4_ + IM_4_ and EP + IM_4_ groups at the primary end point when compared to the ID_0.4_ + IM_4_ group (Fig. 2b). In keeping with the ELISpot data, the EP + IM_4_ group displayed the strongest IFN-ɣ response, with strong MIP-1β and IL-2 responses (Fig. 2c). By contrast in the TC_0.4_ + IM_4_ group higher levels of TNF-α, IL-17a and CD154 were observed. The responses of the ID_0.4_ + IM_4_ group were generally the lowest with the exception of IL-2 and more aligned to the pattern observed in EP + IM_4_ than TC_0.4_ + IM_4_ group. The preferential induction of TNF-α concords with other previous TC studies^16^, while enhanced IL-17a and CD154 expression has not been reported previously. These data suggest a distinct cytokine profile is associated with TC_0.4_ administration when co-administered with large IM_4_ dosage.

A similar trend was observed for CD8 + responses with greater number of responders in the TC_0.4_ + IM_4_ and EP + IM_4_ groups compared to the ID_0.4_ + IM_4_ group (Fig. 2d). The greatest breadth for each vaccinated individual was observed in the TC_0.4_ + IM_4_ and EP + IM_4_ groups compared to the relatively weak responses observed in the ID_0.4_ + IM_4_ group (Fig. 2e). Analysis of the induced cytokine profiles revealed qualitative differences, with EP + IM_4_ inducing the strongest IFN-ɣ responses and TC_0.4_ + IM_4_ responses biased towards TNF-α and IL-2 production. Interestingly while EP + IM_4_ and TC_0.4_ + IM_4_ induced higher levels of granzyme B than ID_0.4_ + IM_4_, the TC_0.4_ + IM_4_ group had elevated levels of perforin expression relative to both ID_0.4_ + IM_4_ and EP + IM_4_.

**Figure 3:**
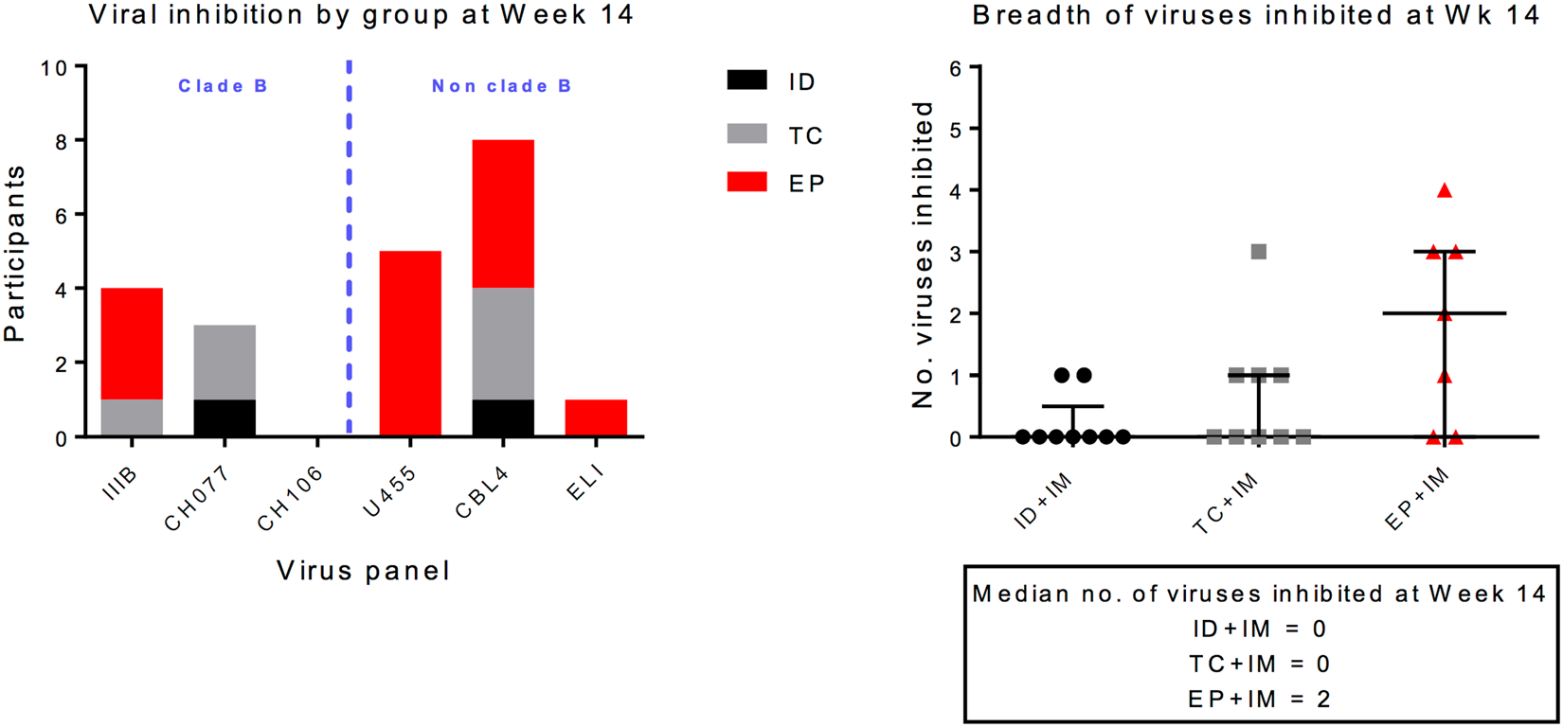
*Ex-vivo* inhibition of HIV replication by CD8 T cells. (**A**) Viral inhibition by group at week 14 showing the number of participants in each group displaying inhibition (positive responders only) and to which virus in the panel. (**B**) Breadth of viruses inhibited at week 14 in each group with median number shown.

Having determined the phenotype of responding cells we evaluated the ability of induced CD8 + cells to inhibit HIV-1 replication in an established viral inhibition assay (VIA) as a functional measure of induced response^28^ (Fig. 3). All groups showed viral inhibitory activity to at least one virus at the primary end point, in 22% (2/9), 44% (4/9) and 71% (5/7) participants across ID, TC and EP groups respectively (Supplemental Table 5). The EP + IM_4_ group also showed the greatest cross clade inhibition (Fig. 3A,B). The inhibitory function of CD8 + T cells appeared to be independent of the cytokine profile (determined by ICS), reflecting the complex interplay of factors that contribute to the overall level of viral inhibition^29,30^. There was also no direct correlation to the kinetics or magnitude of induced T cell IFN-ɣ responses (determined by ELISpot). While one of the two non-responders (C058) in the EP + IM_4_ group had poor T cell responses, the other (C043) responded in a similar fashion to those displaying viral inhibition (Supplemental Fig. 6). Furthermore, there was also no correlation with the number of virus isolates inhibited by positive responders: C036 (4 viruses), C021 and C030 (3 viruses), C049 (2 viruses) and C037 (1 virus) (Fig. 3B) and the magnitude or kinetics of response.

In summary, these data suggest that TC immunization induced a quality of T cell response distinct to EP + IM_4_ and ID_0.4_ + IM_4_.

## Discussion

In this Phase I randomised controlled clinical trial we sought to determine whether the site and mode of DNA vaccination influenced the quality of the induced cellular immune responses. To the best of our knowledge this is the first human study of DNA vaccination by the TC route combined with IM administration. In general, the vaccine and modes of delivery were well tolerated with no serious adverse events reported during the trial. A greater number of local symptoms were reported on the arm following TC administration, including changes in pigmentation in some participants, most likely due to a post-inflammatory response to exogenous DNA^31^ and the process of TC application. Acute, transient injection site pain was associated with EP, but participants considered this acceptable if it provided protection from a serious disease, in line with previous experience using the same device^11,12,32^.

Previous studies in small animals using recombinant protein or human studies using a live attenuated virus have suggested that TC immunization has the potential to induce mucosal IgA in the absence of systemic IgG responses^33,34^. In contrast IM + EP has been associated with induction of systemic antibody responses in humans^20^. The lack of humoral response in any of the treatment groups observed in this study likely reflects the characteristics of the GTU^®^MultiHIV B clade DNA vaccine designed to induce cellular responses. Further work will be required to determine the influence of mode of delivery on humoral response to pDNA vaccination using constructs specifically designed to promote this^20^.

Assessment by conventional ELISpot assay demonstrated that electroporation significantly enhanced IFN-ɣ cellular immune response in terms of magnitude, breadth, and duration of response to different peptides pools. These responses were comparable to that observed in other DNA vaccine trials using EP^11,12^. However a wider phenotypic assessment of the cellular response by ICS revealed a very different response profile. Whereas CD4 + T cell responses were evident in all three groups, these were greater in the TC_0.4_ + IM_4_ and EP + IM_4_ groups compared to the ID_0.4_ + IM_4_ group. The responses were also qualitatively different. EP + IM_4_ induced a predominant IFN-ɣ/MIP-1b/IL2 profile, while TC_0.4_ + IM_4_ administration was biased towards the production of TNF-α/IL-17a/CD154. This alteration in the pattern of CD4 + T cell response is striking given the low dose of DNA applied by TC. These data suggest that TC administration has a qualitatively different impact on induced cellular response to that of ID_0.4_ + IM_4_, where responses were generally lower and, with the exception of IL-2, more closely aligned to EP + IM_4_ than TC_0.4_ + IM_4_.

The preferential induction of TNF-α over IFN-γ responses in the TC_0.4_ + IM_4_ group reflects the phenotype of CD4 + responses seen in a previous clinical trial following the TC administration of the influenza/tetanus vaccine (Tetagrip)^16^. The enhanced expression of IL-17a by responding CD4 + T cells in the TC_0.4_ + IM_4_ group has not previously been reported. IL-17a producing cells (Th17 cells) are thought to play a critical role in host defense against skin and mucosal infection^35,36^, suggesting Th17 cells may be useful targets for vaccine induced mucosal and skin immunity^37^. Although not directly addressed in this study, enhanced expression of CD154 has recently been associated with CD4 + cytolytic activity that is independent of granzyme A/B or perforin expression^38^. This warrants further investigation given the potential cooperative role of HIV-specific cytolytic CD4 + and CD8 + T cells in control of HIV viremia^39^. Together these data indicate that TC-IM DNA administration has a unique immunological profile.

The observation that the magnitude of total ICS CD8 + responses were similar in the TC_0.4_ + IM_4_ and EP + IM_4_ groups relative to the ID_0.4_ + IM_4_ group was unexpected and indicates that TC_0.4_ administration when combined with IM_4_ administration is able to induce a similar magnitude of response to EP + IM_4_. Here again TC_0.4_ + IM_4_ induced stronger TNF-α responses compared to EP + IM_4_ or ID_0.4_ + IM_4_. However, in contrast to previous studies using the Tetragrip vaccine^16^ we saw no evidence for enhanced CD8 + responses associated with TC administration. This likely reflects the difference in vaccine (DNA vs protein) and the combined TC_0.4_ + IM_4_ administration used here in contrast to exclusive TC administration with the Tetragrip vaccine^16^.

Functional assessment of induced CD8 + responses by VIA demonstrated that EP + IM_4_ induced the most potent CD8 + viral inhibition (71% of participants, 5/7 participants) with greater cross-clade activity, impressive in itself given the induced CD8 + responses appeared modest when measured by ICS. One of the two non-responders (C058) in the EP + IM_4_ had poor IFN-ɣ ELISpot responses, however the other (C043) responded in a similar fashion to those showing viral inhibition (Supplemental Fig. 6). Furthermore, there was no correlation between the magnitude or kinetics of response and the number of viruses inhibited. These data suggest that IFN-ɣ ELISpot assays may be a poor predictor of the ability of CD8 + T cells to effect viral inhibition. Nevertheless, the frequency of responders to EP-IM in the viral inhibition was similar to that observed in a previous study delivering an HIV DNA (HIVMAG) vaccine using the same EP device^40^. Interestingly TC_0.4_-IM_4_ induced functional inhibition in 44% of participants in the absence of detectable IFN-γ ELISpot response. The *ex-vivo* ELISpot assay predominantly measures effector memory T cells (Tem) to exogenous peptides. In contrast, the VIA includes an initial *in vitro* expansion of CD8 + cells that would favour the differentiation of central memory T cell (Tcm) responses^41^, associated with durable immune-mediated control in the setting of natural HIV infection^42^. In the context of vaccination, central memory cells can quickly mount greater anamnestic effector responses than those of Tem, which have a limited proliferative capacity and are dependent on persistent antigens. Although there were insufficient cells to determine the antiviral mechanisms or epitope specificity associated with viral inhibition in this study, these will be important issue to address in future studies.

The observation that DNA electroporation enhanced IFN-γ cellular responses that were predominantly CD4 + T cell mediated with a lesser contribution from CD8 + cells supports previous studies^11^. However, the surprising observation was that TC_0.4_ administration when combined with IM_4_ administration, in the absence of EP, induced a total cellular response of similar magnitude but with a distinct cytokine profile to that induced by EP + IM_4_. The higher levels of local reactogenicity and observed changes in pigmentation suggest that DNA by TC administration was capable of inducing local innate responses that might have been critical in shaping the induced immune response. This combined with differences in the number and phenotype of local APC compared to muscle is likely to have caused the strong skewing of the induced cellular response towards a TNFα/IL-17a/CD154 phenotype. Knowing the volume constraints for TC administration (1/10^th^ dose given IM) we chose to evaluate the combined use of TC_0.4_ and IM_4_ administration, therefore we cannot determine the relative contribution of either TC_0.4_ or IM_4_ to the observed immune response profile. Nevertheless, responses to the combination (TC_0.4_ + IM_4_) were greater in magnitude and of a distinct phenotype to that seen with ID_0.4_ + IM_4_ administration. It should be noted that we did not include a group receiving empty plasmid by TC_0.4_ delivery combined with active IM_4_ administration, therefore we cannot exclude the possibility that the innate response to TC_0.4_ DNA delivery alone might have driven a similar response profile. However, previous studies have shown that DNA formulated with cationic polyplexes applied to a large surface area (4 × 40 cm) of exfoliated skin can induce immune response in non-human primates^43^ and are expressed in the hair follicles of mice^18^. Our own data demonstrate that hair follicle targeting following cyanoacrylate skin surface stripping is a promising delivery technique for transcutaneous DNA immunization. Future studies are required to determine the relative contributions of innate and adaptive responses to TC DNA administration and explore potential gains that might be achieved through polyplex formulation^44^, and/or non-invasive skin electroporation^45,46^.

Taken together, these data clearly identify that the site and mode of DNA vaccination can influence the quality of induced cellular responses. DNA given with intramuscular electroporation promoted stronger IFN-γ responses and potent viral inhibition, while transcutaneous administration combined with intramuscular delivery (without EP) shifted responses towards a more Th-17 dominated phenotype, associated with mucosal and epidermal protection. Whilst preliminary, these results offer new perspectives for differential shaping of desired cellular immunity required to fight the wide range of complex and diverse infectious diseases and cancers.

## Materials and methods

### Trial design

CUTHIVAC-001 was a randomised single-site, phase 1 clinical trial in 30 healthy volunteers aged 18–45. Participants were randomised into one of three groups then vaccinated at weeks 0, 4 and 12. The randomisation method was block randomisation using a computer generated algorithm, stratified on gender, and randomisation was carried out within the Inform Electronic Data Capture software on the day of enrolment and first immunisation. Group 1 received ID + IM vaccination, group 2 TC + IM vaccination, and group 3 received IM vaccination with EP delivered immediately after vaccination at the same site using an integrated hand held device. Each volunteer was invited to attend 11 visits over 20 weeks. Vaccinations were performed between May 2014 and February 2015. Each volunteer was followed up for 8 weeks after final vaccination, and the last volunteer completed the study in April 2015. Full details of the trial design are provided in Supplementary Materials (Study Protocol). The trial was conducted in accordance with the clinical trial protocol, the principles of the Declaration of Helsinki, and the International Conference on Harmonization (ICH) Good Clinical Practices standards. Clinical trial authorization was granted by the UK Medicines and Healthcare Products Regulatory Agency. Ethical approval and amendments were granted by the National Research Ethics Service, Cambridge East Research Ethics Committee. Berkshire (ref 13/SC/0023). The trial was registered with the European Union Drug Regulating Authorities for Clinical Trials and assigned the EudraCT No. 2011-003171-11 and was registered under protocol ID ClinicalTrials.gov: NCT02075983 (date of registration 2/27/2014). The trial was performed at the Clinical Trials Centre St Mary’s Hospital, London.

### Study Participants and eligibility criteria

Healthy adults aged between 18–45 at low risk for HIV-1 infection, and with no clinically significant medical history or disorder that presented potential for risk, could influence the results, or impair the participant’s ability to participate in the study were enrolled into the study. Further details of eligibility criteria are set out in the Supplementary Materials (Study Protocol). All participants provided written informed consent.

### Intervention

The investigational GTU^®^MultiHIV B clade DNA vaccine is a synthetic fusion protein comprising of full-length polypeptides of *Rev, Nef, Tat, p17/p24* and *CTL* (containing epitopes of protease, reverse transcriptase and gp160) regions of the HAN-2 HIV B clade virus. This vector developed by FITBiotech is a non-replicating expression vector with enhanced features provided by the bovine papilloma virus transcriptional activators and segregation/partitioning factor E2 protein along with its multimeric specific sites^47^.

### Transcutaneous vaccination

The 0.2 ml (0.4 mg) DNA vaccine was administered by the TC route on the external aspect of the upper left arm below the deltoid muscle over an area measuring 16 cm^2^. A plastic template was used to outline this area using a permanent skin marker (Skin marker H7003 Falc). Shaving was performed on the investigational site as well as on the surrounding skin (2 cm on the top and on the bottom of each investigational site and 1 cm on both sides) using a dry razor (Disposable razor, Art.-No. 182 H, Wilkinson Sword). After shaving, CSSS was performed as described elsewhere^14^. In brief, a total amount of 190 mg (equivalent to 9 drops) of cyanoacrylate glue (Superglue, UHU KG) was applied over the investigational site. A microscope slide was used to spread the glue evenly on the skin surface, followed by the application of adhesive tape (6 × 5 cm, Art.-No. 571176–00000, Tesa Beiersdorf) which was rolled with a rubber roll over a sheet of paper on the investigational site to improve adherence (10 times). After hardening of the glue for 20 min, the tape and glue were removed from the skin surface. In order to prevent uncontrolled spreading and loss of vaccine, a rectangular silicone frame (inner surface 5 × 3.2 cm, ModulorGmbH, Berlin, Germany) was taped onto the investigational site. The vaccine was then applied drop-wise from the original syringe as provided by the manufacturer (16 drops of <16 μl each) followed by a soft massage. This was performed using a finger cot pre-soaked (for 20 mins) in DNA vaccine (care&serve, Wiros) and massaging the DNA in small circles for 1 minute over the investigational site. This application procedure was followed by an incubation time of 20 min after which a hydrocolloid bandage (Comfeel Plus Transparent 9 × 14 cm, Art.-No. 3542, Coloplast A/S) was placed over the arm for 24 h. The volunteers were instructed not to take a shower or bath and to avoid any activity that may cause sweating or mechanical stress to the investigational sites (e.g., physical exercise) during these 24 hours.

### Intradermal injection

DNA vaccine (0.4 mg) as provided by the manufacturer was administered as 2 × 0.1 ml (0.2 mg) injections ID via a small needle (25 or 27 gauge), inserted into the dermis of each upper arm below the deltoid muscle. The ‘bleb’ or weal created was used as a marker of accuracy and to ensure consistency of injection into the intradermal layer.

### Intramuscular injection

DNA vaccine (4 mg) as provided by the manufacturer was injected administered as 2 × 1 ml (2 mg) injections IM via a needle, with 1 ml into each vastus lateralis muscle of the upper thigh at every immunization time point.

### Intramuscular injection with electroporation

The disposable electroporation cartridge was loaded with the DNA vaccine and then adjusted to one of three depth settings, corresponding to pre-defined ranges in skin fold thickness. The specified dose (4 mg) required bilateral administration of 1 ml (2 mg) at each vaccination time into each vastus lateralis muscle of the upper thigh. The cartridge was loaded into the EP device and applied to the muscle. IM administration was followed immediately by the application of electrical stimulation (TriGrid^TM^ Delivery System, Ichor Medical Systems, San Diego, CA). The spacing of the TriGrid^TM^ electrode array was 6 mm in a diamond-shaped configuration, and the electrical field was applied at an amplitude of 250 V/cm of electrode spacing for a 40 millisecond total duration applied as three pulses over a 400 millisecond interval, resulting in brief muscle contractions. All electroporation procedures were performed by trained members of the clinical team.

### Objectives and primary end-point measures

The main objective was to investigate the safety and immunogenicity of the three different routes of administering DNA- GTU^®^ MultiHIV B clade vaccine (ID + IM, TC + IM, and EP + IM). Oversight for the trial was provided by the Trial Management Group with the option to refer any notable events to the IDMC. The primary safety endpoint was a grade 3 or above solicited local, general or laboratory adverse event or any event leading to a clinical decision to discontinue immunisations.

The primary immunogenicity endpoint was the presence of a T cell response measured by IFN-γ ELISPot assay using frozen PBMCs collected 2 weeks after the final immunisation. Immunogenicity assays are detailed below, and sample processing and analyses of all immunology data were performed observer-blinded by use of a randomly generated laboratory identifier.

### Reactogenicity and safety

Participants returned for a 24 hour and 7 day post vaccination safety visit, where they were monitored for local and systemic reactogenicity and adverse events. In addition, participants filled out diary cards from 12 hours to 7 days post vaccination. The frequency and severity of solicited and unsolicited local and systemic adverse events starting within 1 week after vaccination were assessed together with safety bloods (full blood count and differential, renal and liver biochemistry), and reported in the electronic database. Adverse events were graded using modified US Food and Drug Administration and Division of AIDS criteria. Those randomised to EP + IM were asked to fill out a pain questionnaire rating their experience of the procedure, using a visual analog pain score (0 = no pain, 10 = worst pain ever), and to complete an acceptability questionnaire.

### IFN-γ ELISpots assay

IFN-γ ELISpot assays were performed using frozen isolated peripheral blood mononuclear cells (PBMCs) stimulated with peptide pools matched to the GTU^®^MultiHIV B vaccine, at weeks 0, 1, 4, 5, 8, 12, 14 (primary end point) and 20. In brief, frozen PBMCs at a concentration of 1 × 10^7^ were rested overnight and resuspended to a final concentration of 4 × 10^6^ viable PBMC/ml. 50 µl cells/well in triplicate were added to 96 well plates with an additional 50 µl stimulation media containing peptide pools *Rev, Tat, Nef, Gag, CTL* with 2 positive controls (*PHA and CEFT*) at a final concentration of 5 µg/ml (2.5 µg/ml in the wells). Negative controls wells were media alone plus cells. Plates were incubated for 16–24 hours at 37 °C/5% C0_2_. Plates were washed with sterile PBS then incubated for 2 hours at room temperature with primary antibody (mouse-anti human IFN-ɣ at a. concentration of 1 µg/ml). The signal was amplified with 1 hr incubation with streptavidin-ALP solution, then developed with substrate (BCIP/NBT) for between 5–7 minutes, stopped by washing with cold water and allowed to dry overnight in the dark, before being read with an automated AID iSPOT ELISpot plate reader

The number of spot-forming units per million cells or SFU/M, was calculated as the mean count minus the background count. A positive response was defined according to two criteria 1) The positive control was >1000 SFU/M; 2) The mean of the negative wells was <100 SFU/M; 3) The positive response was >4 x mean background (or greater than 0 if the background was 0) and >55 background subtracted SFU/M^48,49^.

### Intracellular cytokine analysis

T cell responses evaluated by intracellular cytokine staining (ICS) with two staining panels for CD8 + and CD4 + responses. In brief, cryopreserved PBMC were thawed, rested overnight in R10 media at 37 °C/5% CO_2_ prior to stimulation with vaccine matched peptide pools and PMA /Ionomycin (positive control) or were unstimulated (negative control). After incubation, cell viability was assessed, 1 × 10^6^ viable PBMC were incubated for 6 hours at 37 °C stimulated with 2.5 µg/mL of each peptide pool (*Nef, Rev, Gag, Tat* or *CTL*) plus 1 µg/mL CD28/49d (BD Biosciences). Two hours into the stimulation, Brefeldin A was added. Cells were stained with fixable viability dye eFluor780 (eBioscience, UK), CD3 BV650, CD4 PerCP-Cy5.5, CD8 AF700 (BioLegend, San Diego, CA), CD14 APC-Cy7 (BD Biosciences, UK) IFN-γ AF488, TNF-α PE-Cy7, IL-2 BV510, IL-17A AF647, CD154 BV421, Perforin PE, Granzyme B Pacific Blue (BioLegend) and MIP-1β PE (BD Biosciences) in two panels to assess CD4/CD8 specific responses. Cells were fixed and stored at 2–8 °C before analysis on a Becton Dickinson FortessaLSRII equipped with 50 mW 405 nm, 50 mW 488 nm, 50 mW 561 nm, 20 mW 633 nm lasers and a ND1.0 filter in front of the FSC photodiode. Acquisition was performed on live gated populations with 50,000 events CD3 + lymphocytes after dead cell and doublet exclusion (FSC-A/W, SSC-A/W gating), using FlowJo v10 software (Treestar, Ashland). Data was expressed as percentages of total CD4 + or CD8 + cells. Background responses in negative controls un-stimulated were subtracted from the stimulated samples. Responders were defined as having a percentage staining greater than 0.05% and with a response greater than 3 x the baseline prior to vaccination.

### Humoral Immunogenicity

The level of binding antibodies against 4 different HIV-1 clade B recombinant proteins (*rev, tat, nef and gag*) encoded in the vaccine were determined using a standard indirect ELISA protocol. Briefly, ELISA plates (Greiner Bio-One, medium binding, 96-well flat bottom) were coated with 1 μg/mL of each of the HIV-1 recombinant proteins (provided by the Centre for AIDS Reagents, NIBSC UK) or 5 μg/mL anti-human kappa/lambda antibody (Southern Biotech, USA) for standard curve capture, in assay buffer (PBS with 1% BSA and 0.05% Tween 20) for 1 hour at 37 °C. After blocking, and washing, sera diluted 1:100 in assay buffer from baseline, weeks 1, 4, 5, 12, 14 and 20 were incubated for 1 hour at 37 °C. Standard curves were prepared starting at 1 µg/mL for purified IgG and 5 µg/mL for IgA (Sigma Adrich, UK) and serially diluted 1:5. Plates were washed 4 times with PBST (PBS with 0.05% Tween 20). Goat anti-human IgG HRP- or IgA HRP-conjugated antibodies (Sigma Aldrich, UK) were used to detect antigen specific IgG and IgA antibodies. Substrate was added (SureBlue TMB 1-Component peroxidase substrate KPL, UK) and stopped after 5 minutes with the addition of TMB stop solution (KPL, UK). Plates were then read at 450 nm on a VersaMax 96-well microplate reader (Molecular Devices). The ELISA data is expressed as positive if the blank-subtracted OD_450__nm_ was above the pre-determined cut-off of O.D 0.2 nm and values were on the linear range of the standard curve. To ensure assay sensitivity, a positive control composed of pooled plasma samples from HIV positive donors was used. Analysis of the data was performed using SoftMax ProGxP software (version 5.4, Molecular Devices). Mucosal samples were also collected at baseline and the primary end point. Genital tract secretions from women were collected using the Instead Softcup™ (Evofem Inc) and urethral swabs (Hunt Biologics, UK) from male volunteers and rectal Floq™ swabs were taken when possible from males and females, primarily to assess the feasibility of the sampling method. Vaginal samples were self-inserted by female participants, collected after 60 mins, processed and analysed as described previously^50^. Urethral swabs were self-inserted by male participants in clinic by inserting the swab into the urethra allowing it to absorb secretions for 2 minutes. Rectal Floq™ swabs were clinician-inserted into the anus and rotated to collect secretions, either with or without the use of a proctoscope depending on participant preference. Rectal and urethral swabs were either snap frozen on receipt or processed directly. Rectal and urethral swabs were placed in the top chamber of a spin X tube and exudates either analysed immediately using the ELISA method described above, or were aliquoted and frozen at −80 °C until analysis.

### Viral Inhibition Assay

This assay is based on a modified version of a previously established assay^28^. Two time points were assessed for the presence of viral inhibitory activity: a pre-vaccination time point (week 0) and the primary end point (week 14, two weeks after the third and final vaccination). Therefore for each participant, there was generation of one culture of pre vaccination CD4 + T cells and two CD8 + T cell cultures (one pre and one post vaccination). A panel of six viruses was used in this assay. Three clade B viruses were used in the panel, (IIIB, CH077, CH106) with 3 cross clade viruses (ELI clade AD, U455 clade A, CBL4 clade D). HIV-1 isolates IIIB (accession number HXB2 K03455, subtype B), ELI (K03454, subtype A/D) and U455 (M62320 subtype A), were provided by the HIV AIDS reagent repository, CH77 (JN944909, subtype B) and CH106 (JN944897, subtype B) were generously donated by George Shaw, University of Birmingham, AL, USA) and CBL4 (subtype D) was provided by the National Institutes of Biological Standards and Control, UK).

Frozen PBMCs from the specified time points were thawed and adjusted to 0.8–1.5 million viable PBMC per ml with R10 media. For the generation of CD4 + targets, PBMC were incubated for 3 days (37 °C/5% C0_2_) in R10 media supplemented with 0.5 µg/ml CD3/8 bispecific antibody (kindly provided by J. Wong, Harvard Medical School) and 50 units/ml IL-2 (Roche). For CD8 + cells, PBMCs were cultured in R10 supplemented with 0.5 µg/ml CD3/4 bispecific antibody (J. Wong) and 50 units/ml IL-2. On day 3 the starting volume was doubled by adding R10/50 (50 units IL-2 per ml of R10). On day 6, the culture volume was again doubled and incubation continued. On day 7 (day of infection), cells were re-suspended and counted. CD4 + cells were infected with the virus panel specified at a multiplicity of infection (MOI) of 0.01 (10000 TCID_50_ per million CD4 cells) for 4 hours at 37 °C/5% C0_2_. Cells were washed once and 0.5 × 10^6^ infected CD4 T cells cultured with 0.5 × 10^6^ expanded CD8 T cells from baseline (week 0) or the primary end point (week 14) in 1 mL of R10/50 medium in 48-well plates. Post infection on days 3, 6, 8, and 10, 500 µl supernatant was removed and replaced with fresh R10/50. On day 13 post infection supernatant was removed and virus lysed using 10% triton (Perkin Elmer) and frozen. The supernatants were analysed for p24 content at a later stage using an ELISA (Perkin Elmer) and CD8 + T cell mediated inhibition was indicated by the level of p24 in the supernatant of infected cells plus CD8 + cells from the time points, in comparison to infected CD4 + cells alone. The VIA response for each virus was considered positive if the difference between post and pre vaccination time points was ≥0.6 log^10^ inhibition, and above the 97.5^th^ percentile defined as >1.5 log^10^ inhibition.

### Statistical analysis

All clinical and routine laboratory data were included in the safety analyses. Data sets included: (i) intention to treat (ITT); all participants who were randomised and received at least one vaccination and (ii) per protocol (PP); all participants who completed vaccinations with no major protocol deviations. The primary safety outcome was expressed as a proportion of participants with 95% confidence interval, and groups were compared using Fishers exact test. There was no pre-specified hypothesis on which to power the study. Immunological analyses were based on the PP population that received all vaccinations. Appropriate comparative statistics are annotated in the text. A two-sided P value of <0.05 was considered statistically significant and data were analysed using Graphpad Prism software version 6.0. For the immunogenicity analysis, the primary end point was defined as 2 weeks after the third and final vaccine (week 14). The difference in magnitude of the INF-ɣ response between the groups at the specified time points including the primary end point for each peptide was compared using an unpaired non-parametric t test (Mann-Witney test). Results were considered statistically significant if the 2 tailed P value was <0.05. For the analysis of the difference within each individual group week 0 and week 14, a paired t test (Wilcoxon matched-pairs singed rank test) was used where statistical significance was set at p = <0.05.

### Data Availability

All data generated or analysed during this study are included in this published article (and its Supplementary Information files). Raw data files are available from the corresponding author on reasonable request.

## Electronic supplementary material

Consort 2010 Checklist

Supplementary Information
