# Supplementary material for: Combined skin and muscle vaccination differentially impact the quality of effector T cell functions: the CUTHIVAC-001 randomized trial
Source: Sci Rep. 2017 Oct 12;7:13011. doi: 10.1038/s41598-017-13331-1 (PMC5638927; doi:10.1038/s41598-017-13331-1)
Supplement: Supplementary file 2 — Supplementary Information [file 41598_2017_13331_MOESM2_ESM.pdf]

Supplementary Information Title: **Combined skin and muscle vaccination differentially impact the quality of**

effector T cell functions in humans: the CUTHIVAC-001 randomized trial

Authors G Haidari[1], A Cope[1], A Miller[1], S Venables[1], C Yan[1], H Ridgers[1], K Reijonen[2], D Hannaman[3], A Spentzou[1], P Hayes[4], G Bouliotis[1], A Vogt[5], S Joseph[6], B Combadiere[7], S McCormack[6], RJ Shattock[1]

Supplemental Table 1: Trial inclusion and exclusion criteria

INCLUSION CRITERIA	EXCLUSION CRITERIA
Men and women aged between 18-45	Pregnant or lactating
BMI between 19-30	Use of topical treatment on injection or application site within last 4 weeks
Willing and able to given informed written consent	No UV tanning sessions or strong sun exposure within 4 weeks prior to study
At low risk of HIV and willing to remain so for the duration of study defined as:<br>• No history injecting drug use in last 10 years<br>• No gonorrhoea or syphilis infection in last 6 months<br>• No 'high risk' partner (eg IDU, HIV positive) currently or in last 6 months<br>• No UPAI in last 6 months outside of relationship with regular HIV negative partner<br>• No UPVI in last 6 months outside of relationship with regular HIV negative partner	Clinically relevant abnormality on history or examination including:<br>• History of seizure disorder<br>• Severe eczema<br>• Liver disease with inadequate hepatic function<br>• Any skin condition which may interfere with injection site<br>• Haematological, metabolic, gastrointestinal, cardiac disorders including abnormal ECG<br>• Autoimmune disease or use of immunosuppressives in last 3 months
If heterosexually active female using an effective method of contraception (includes consistent condom use), if heterosexually active male using effective method with partner	Excessive terminal hair growth on investigational areas
	Grade 1 or above abnormal laboratory parameters (including conjugated hyperbilirubinaemia)
Agree to abstain from blood donation for 3 months after the end of trial or longer if necessary	Skin fold measurement on upper thighs >40mm
	HIV-1 or 2 positive, HepBsAg positive, HepC Ab positive
	Previous severe reaction to vaccination or receipt of live attenuation vaccine in last 60 days

Supplemental Figure 1: CONSORT participant flow diagram

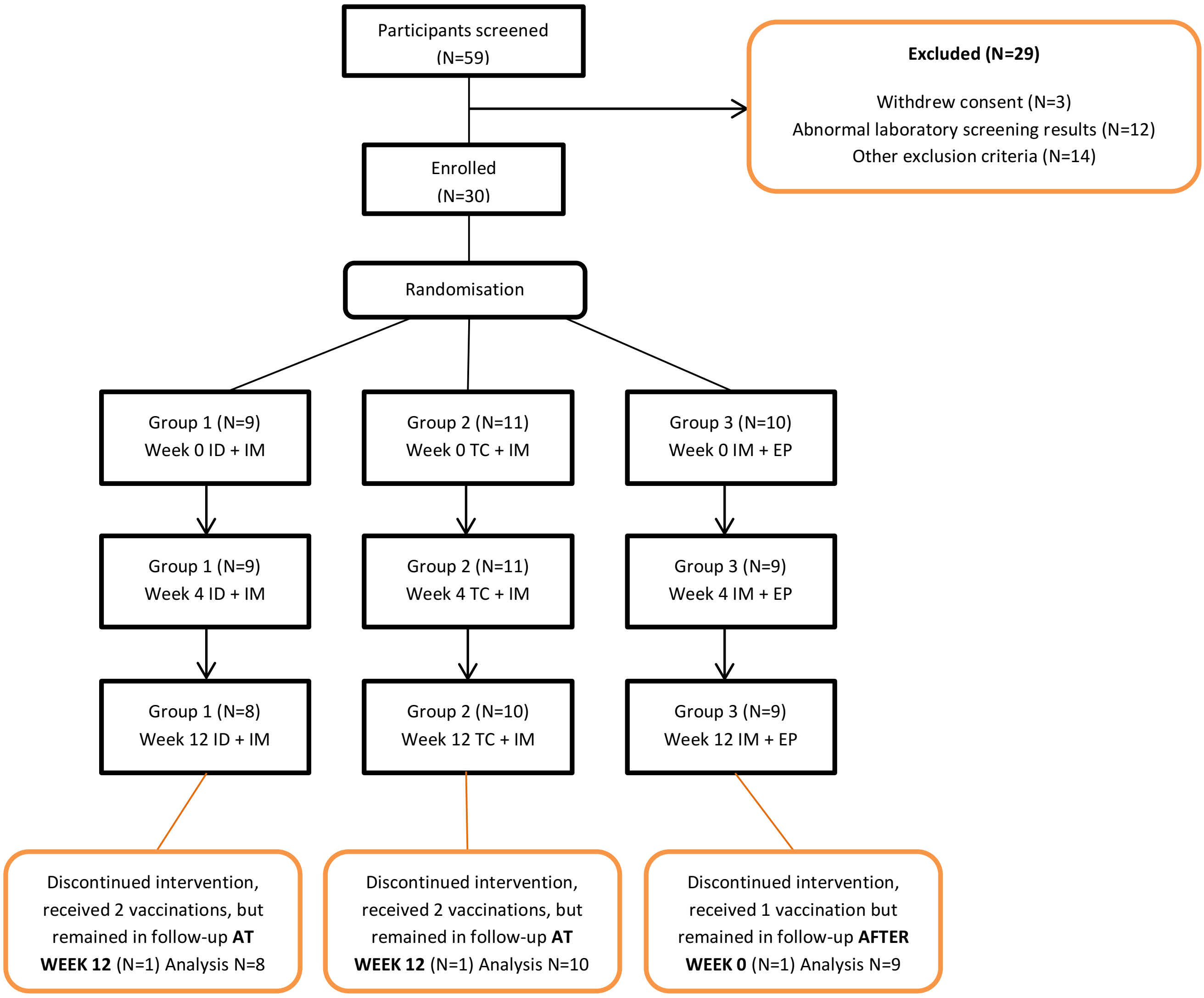

Supplemental Table 2: Participant demographics of all enrolled (N=30)

**Demographics**	Group 1 (ID + IM)	Group 2 (TC + IM)	Group 3 (EP + IM)	**Total**
**Gender**				
Male	5	6	6	**17**
Female	4	5	4	**13**
**Ethnicity**				
White British	6	2	4	**12**
White Other	2	5	4	**11**
Mixed Ethnic Group	1			**1**
Black African		1	1	**2**
Black Other		1	1	**2**
Asian (Chinese)		1		**1**
Asian (Other)		1		**1**
**Age (years)**				
Mean	28.6	31.5	31.7	**30.7**
Range	(22-34)	(21-42)	(23-39)	**(21-42)**
**Employment status**				
Full time	7	7	7	**21**
Part time			1	**1**
Unemployed	1			**1**
Student	1	4	2	**7**

Supplemental Table 3: Unsolicited adverse events showing relationship to vaccine (determined by clinical team). All unsolicited adverse events were Grade 1 or Grade 2.

**Safety: Adverse events**	**Group 1 ID+IM**	**Group 2 TC+IM**	**Group 3 EP+IM**	**Total**
Definitely related to vaccine	0	0	1	1 (2%)
Possibly related	6	3	0	9 (15%)
Probably related	0	4	0	4 (7%)
Unlikely related to vaccine	20	14	12	46 (76%)
**Total**	**26**	**21**	**13**	**60 (100)**

Supplemental Figure 2: Solicited systemic and local reactogenicity from participant diary cards. General symptoms documented by participants on their diary cards filled out from D0 to D7 post vaccination. Cumulative results shown from all 3 diary cards where y axis is the number of events and x axis reflect symptoms. B: Local arm symptoms, ID+IM and TC+IM groups only as EP+IM did not receive vaccination to any arm. C: Local leg symptoms documented from all 3 groups where ID+IM and TC+IM groups received IM injection to upper thigh and EP+IM received IM injection followed by EP using the integrated device.

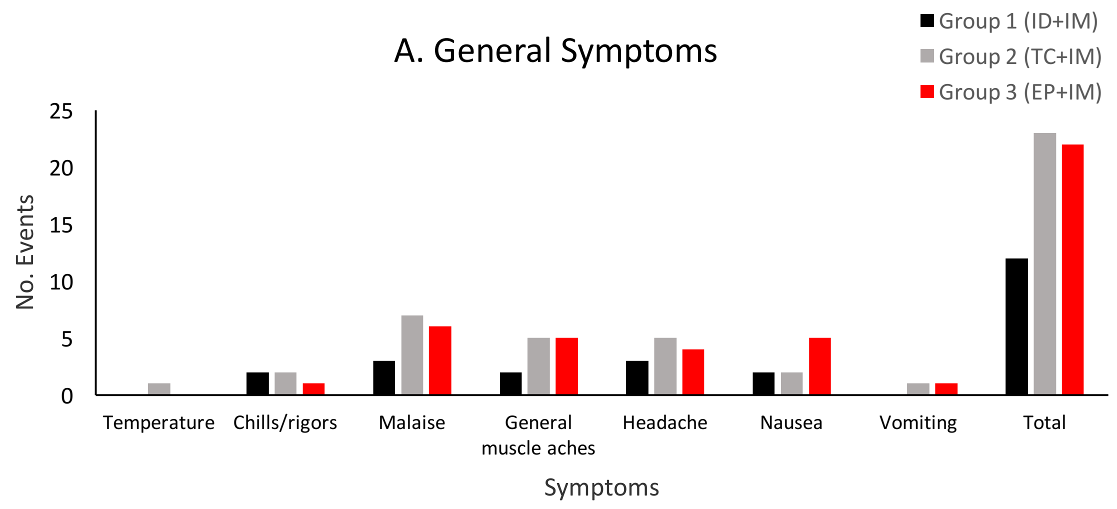

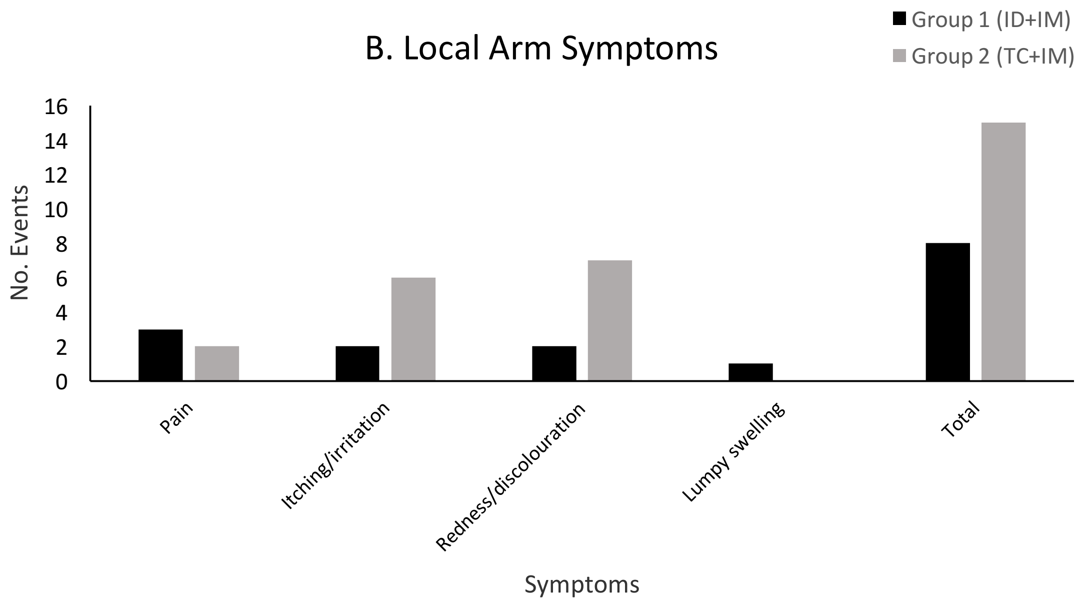

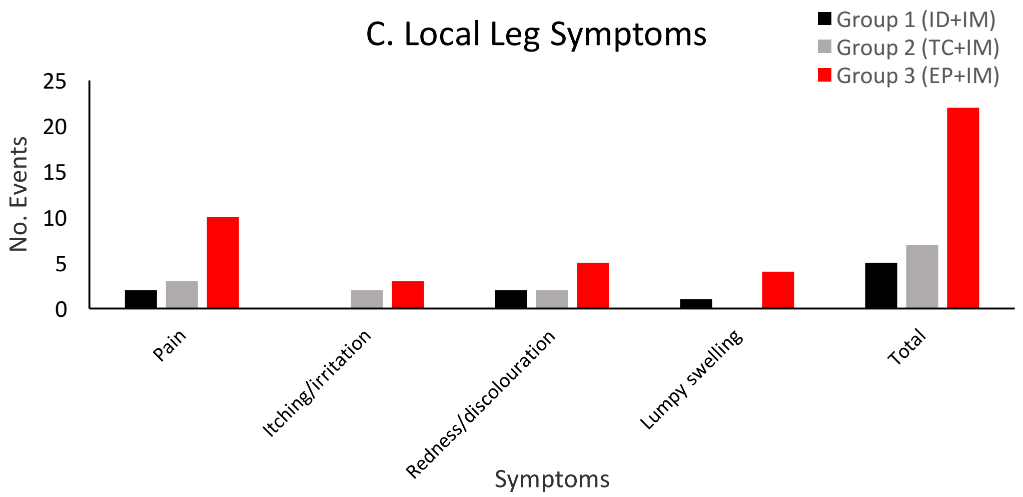

C. Local Leg Symptoms
Group 1 (ID+IM)
Group 2 (TC+IM)
Group 3 (EP+IM)
No. Events
25
20
15
10
5
0
Pain
Itching/irritation
Redness/discolouration
Lumpy swelling
Total
Symptoms

Supplemental Table 4: Summary of solicited adverse events from participant diary cards. All events were either grade 1 or grade 2 (mild/moderate) with no grade 3 or 4 reactions documented from participant diary cards. The numbers documented represent the number of participants documenting the symptom at any time point. Therefore if participants documented the same symptom on every diary card, this is only counted once. For local arm reactogenicity, there are no recording for the EP+IM group as this group did not receive vaccination to any arm.For local leg reactogenitiy the ID+IM and TC+IM groups received intramuscular vaccination to each leg, whereas the EP+IM group received IM vaccination followed by EP at the same site to each leg. Statistical significance was set at p=<0.05

	Symptoms	ID+IM (Group 1) (n=9)	TC+IM (Group 2) (n=11)	EP+IM (Group 3) (n=10)
Systemic reactions (p=0.49)	Temperature	0	1	0
	Chills/Rigors	2	2	1
	Malaise	3	7	6
	General muscle aches	2	5	5
	Headache	3	5	4
	Nausea	2	2	5
	Vomiting	0	1	1
	**Total**	**12**	**23**	**22**
Local reactions Arm (p=0.69)	Pain	3	2	n/a
	Itching/irritation	2	6	
	Redness/discolouration	2	7	
	Lumpy swelling	1	0	
	**Total**	**8**	**15**	
Local reactions Leg (p=0.03 ID vs EP) (p=0.06 TC vs EP)	Pain	2	3	10
	Itching/irritation	0	2	3
	Redness/discolouration	2	2	5
	Lumpy swelling	1	0	4
	**Total**	**5**	**7**	**22**

Supplemental Figure 3: Tolerability questionnaire for EP. Cumulative responses from pain questionnaire filled out by participants in this group after every vaccine procedure. Participants were asked to rate their pain out of 10 (1-3=mild; 4-7=moderate; 8-10=severe). Responses shown are cumulative and the maximum response is taken for both legs to give one final response.

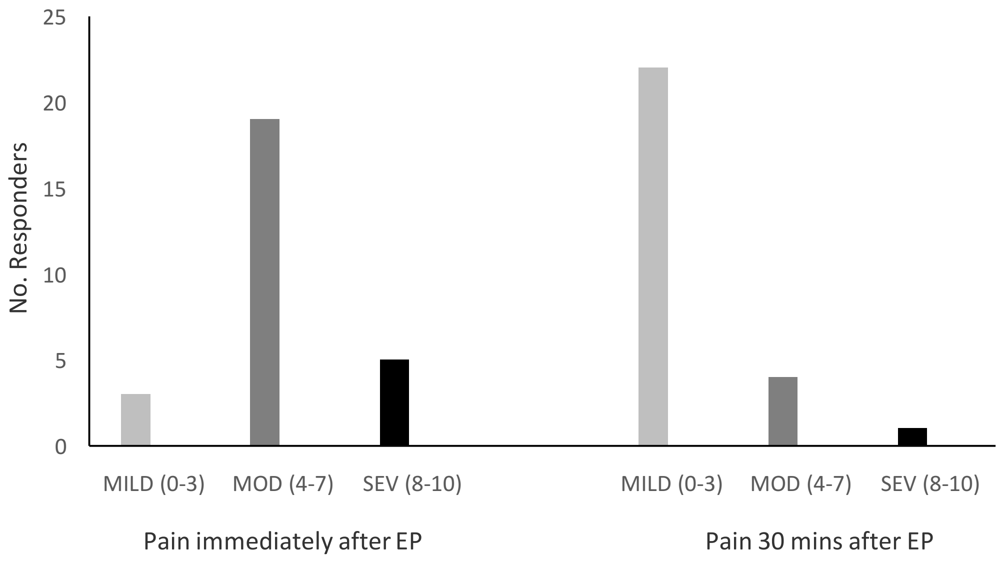

Supplemental Figure 4: Acceptability questionnaire for EP. Question 1 where y axis is percentage of responders voting yes, no or not sure. Question 2 where y axis is percentage of responders voting yes, no or not sure.

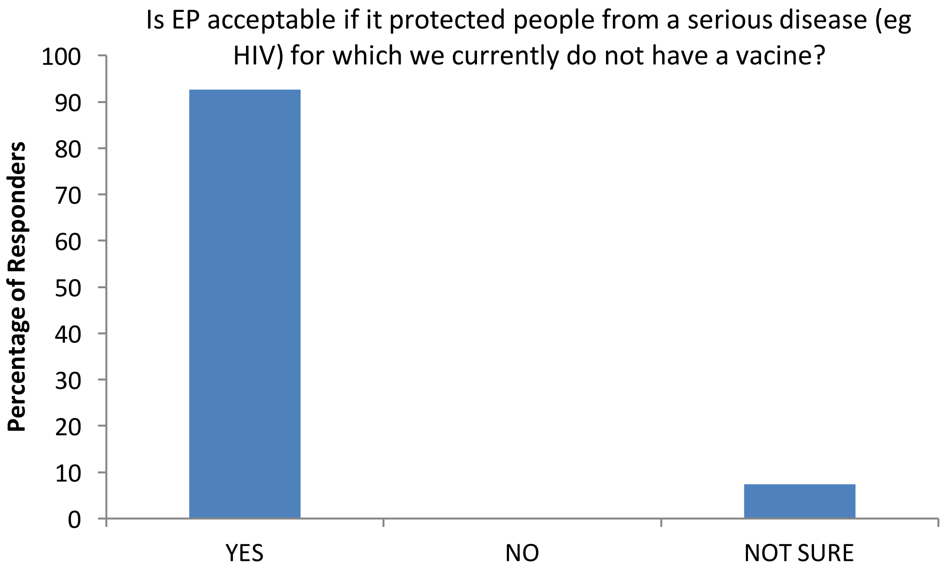

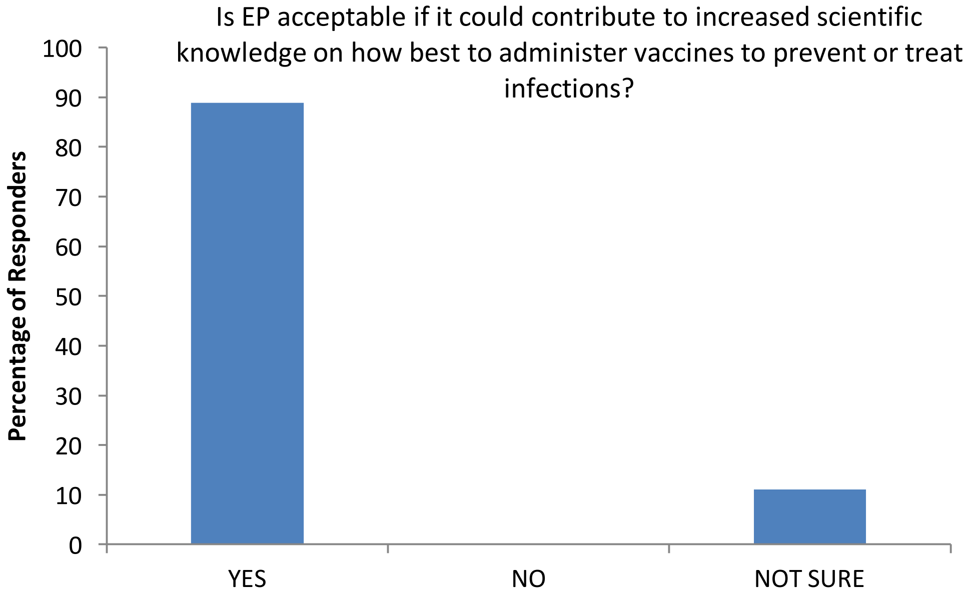

Supplementary Table 5: IFN-ɣ responses at week 14 shown by group and per peptide with mean, median and p values shown. Statistical significance is set at p=<0.05.

	Group 1<br>ID+IM	Group 2<br>TC+IM	Group 3<br>EP+IM	**Total**<br>**(%)**
**IFN-ɣ response**<br>**at primary end point (n/total)**	1/9 (11%)	0/11 (0%)	9/10 (90%)	10/30 (33%)
**Rev (SFU/M)**				
Mean (SEM)<br>Median<br>25-75 percentile	10 (3)<br>8<br>4-16	3 (2)<br>0<br>0-5	120 (39)<br>91<br>12-190	TC+IM vs ID+IM **p=0.0120**<br>EP+IM vs ID+IM **p=0.0182**<br>EP+IM vs TC+IM **p=0.0005**
**Tat (SFU/M)**				
Mean (SEM)<br>Median<br>25-75 percentile	9 (3)<br>8<br>1-13	7 (3)<br>7<br>0-12	35 (10)<br>23<br>6-77	TC+IM vs ID+IM p=0.4547<br>EP+IM vs ID+IM p=0.0811<br>EP+IM vs TC+IM **p=0.0229**
**Nef (SFU/M)**				
Mean (SEM<br>Median<br>25-75 percentile	30 (8)<br>28<br>11-49	14 (4)<br>10<br>0-25	212 (45)<br>216<br>91-311	TC+IM vs ID+IM p=0.1455<br>EP+IM vs ID+IM **p=0.0007**<br>EP+IM vs TC+IM **p=<0.0001**
**Gag (SFU/M)**				
Mean (SEM)<br>Median<br>25-75 percentile	28 (18)<br>10<br>1-25	4 (3)<br>0<br>0-7	189 (49)<br>133<br>67-317	TC+IM vs ID+IM p=0.0737<br>EP+IM vs ID+IM **p=0.0014**<br>EP+IM vs TC+IM **p=<0.0001**
**CTL (SFU/M)**				
Mean (SEM)<br>Median<br>25-75 percentile	19 (6)<br>7<br>3-41	16 (5)<br>7<br>0-35	116 (42)<br>75<br>23-187	TC+IM vs ID+IM p=0.3518<br>EP+IM vs ID+IM **p=0.0109**<br>EP+IM vs TC+IM **p=0.0063**

Supplementary Figure 5: Duration of IFN-ɣ response across entire trial and out to week 20 shown by group.

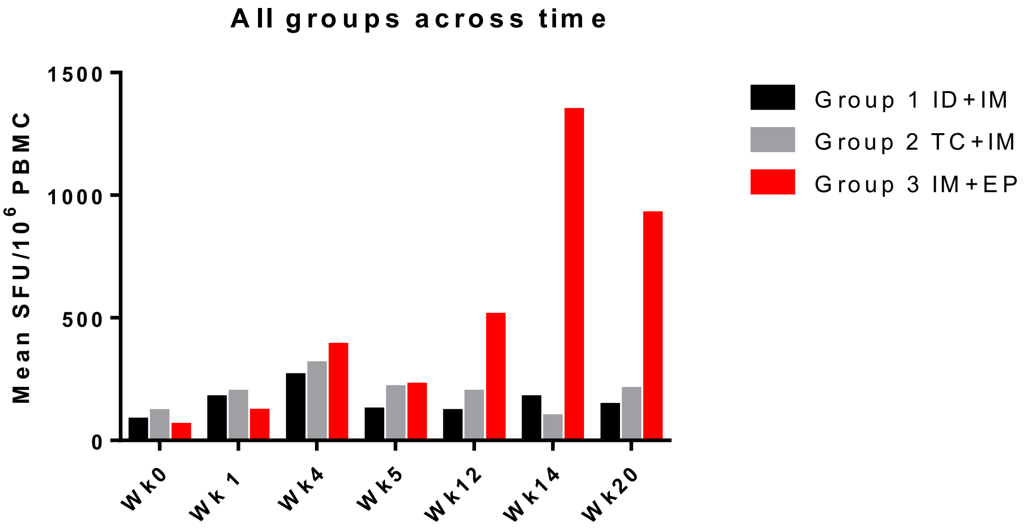

Supplemental Table 6: Table to show the number of participants meeting the criteria for viral inhibition, with median and maximum log^{10} inhibition shown.

	**ID+IM**	**TC+IM**	**EP+IM**
No. Volunteers	9	9	7
Median Log^{10} Inhibition	1.09	1.01	1.42
Max Log^{10} Inhibition	1.90	2.31	2.88
VIA Responders	2/9	4/9	5/7
% Response rate	22.2	44.4	71.4

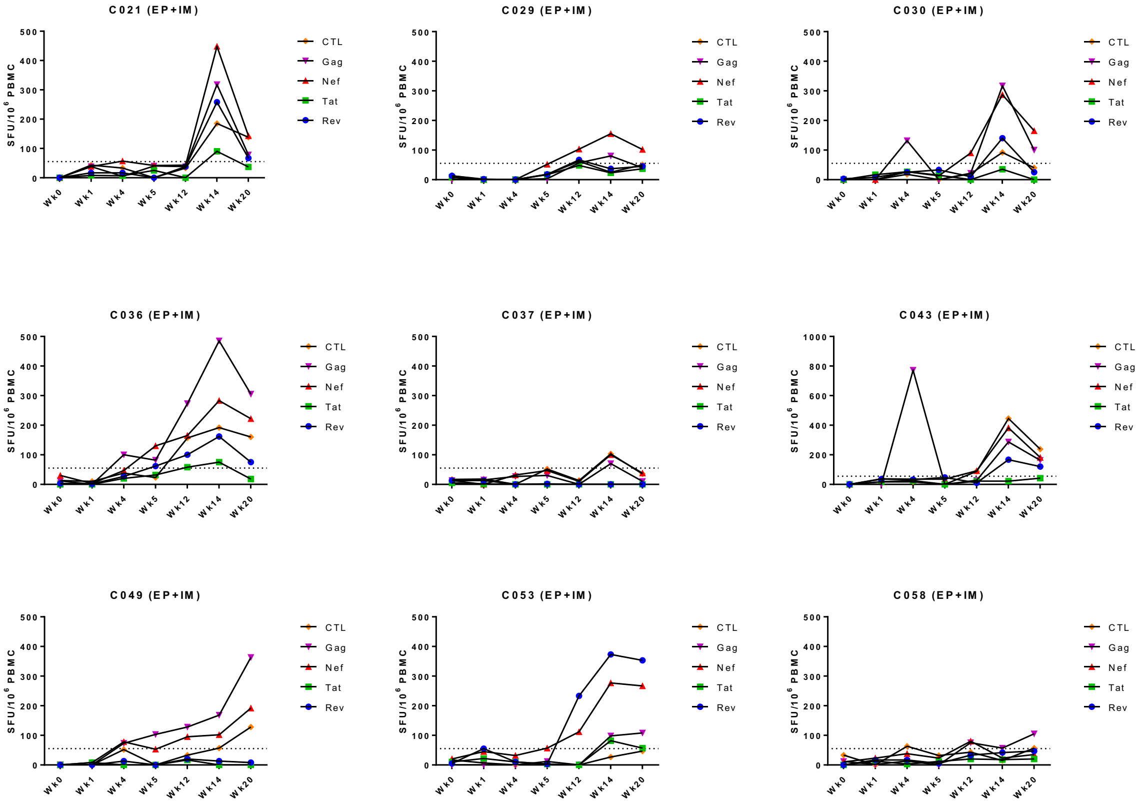

Supplementary Figure 6: Individual IFN-ɣ responses from participants in the EP+IM group to show T cell responses to peptide pools by week. All values are in spot forming units per million PBMC (SFU/10^6).
